# Supplementary material for: Increasing countries’ financial resilience through global catastrophe risk pooling
Source: Nat Commun. 2023 Feb 17;14:922. doi: 10.1038/s41467-023-36539-4 (PMC9938104; doi:10.1038/s41467-023-36539-4)
Supplement: Supplementary file 1 — Supplementary Information [file 41467_2023_36539_MOESM1_ESM.pdf]

Supplementary Information

Increasing countries' financial resilience through global catastrophe risk pooling

Alessio Ciullo [1,2]*, Eric Strobl [3], Simona Meiler [1,2], Olivia Martius [4], David N. Bresch [1,2]

[1] Institute for Environmental Decisions, ETH Zurich, Zurich, Switzerland
[2] Swiss Federal Office of Meteorology and Climatology MeteoSwiss, Zurich, Switzerland
[3] Department of Economics and Oeschger Centre for Climate Change Research, University of Bern, Switzerland
[4] Institute of Geography and Oeschger Centre for Climate Change Research, University of Bern, Switzerland

* Corresponding author, alessio.ciullo@usys.ethz.ch

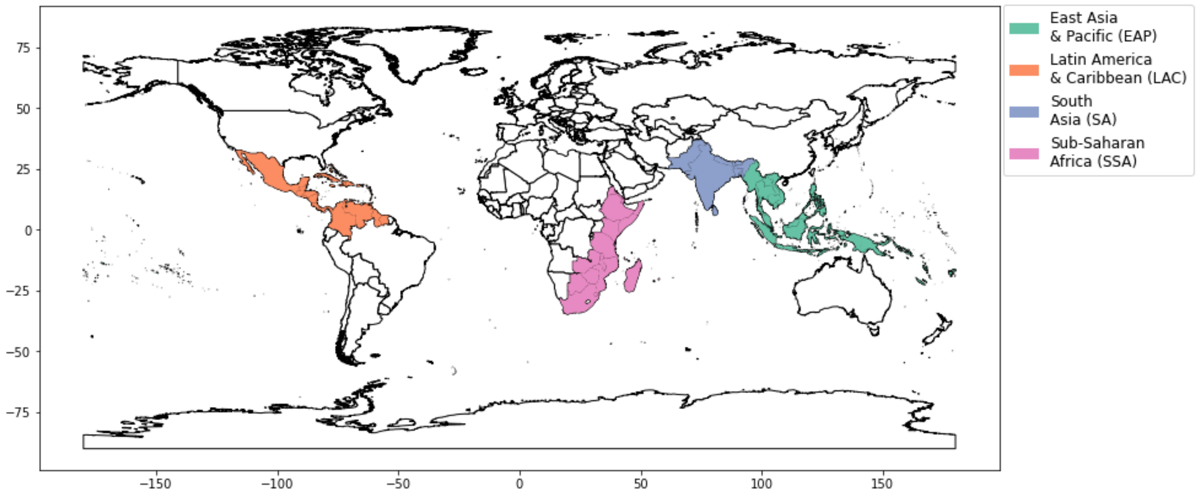

Figure S1 The considered four geographical regions: East Asia & Pacific (EAP), Latin America & Caribbean (LAC), South Asia (SA) and Sub-Saharan Africa (SSA). Regions are identified following the World Bank's official regional classification and retaining only middle- to low-income countries facing tropical cyclone risk.

Table S1 ISO 3166-1 alpha-3 codes and countries' names for the East Asia & Pacific (EAP) region.

ISO3 Code	Name of Country
ASM	American Samoa
COK	Cook Islands
FJI	Fiji
FSM	Federated States of Micronesia
IDN	Indonesia
KHM	Cambodia
KIR	Kiribati
LAO	Laos
MHL	Marshall Islands
MMR	Myanmar
MYS	Malaysia
NCL	New Caledonia
NIU	Niue
PHL	Philippines
PLW	Palau
PNG	Papua New Guinea
PYF	French Polynesia
SLB	Solomon Islands
THA	Thailand
TLS	East Timor
TON	Tonga
TUV	Tuvalu
VNM	Vietnam
VUT	Vanuatu
WLF	Wallis and Futuna
WSM	Samoa

Table S2 ISO 3166-1 alpha-3 codes and countries' names for the Latin America & Caribbean (LAC) region.

ISO3 Code	Name of Country
ABW	Aruba
AIA	Anguilla
ATG	Antigua and Barbuda
BHS	The Bahamas
BLM	Saint Barthelemy
BLZ	Belize
BMU	Bermuda
BRB	Barbados
COL	Colombia
CRI	Costa Rica
CUB	Cuba
CUW	Curaçao
CYM	Cayman Islands
DMA	Dominica
DOM	Dominican Republic
GRD	Grenada
GTM	Guatemala
GUY	Guyana
HND	Honduras
HTI	Haiti
JAM	Jamaica
KNA	Saint Kitts and Nevis
LCA	Saint Lucia
MAF	Saint Martin
MEX	Mexico
MSR	Montserrat
NIC	Nicaragua
PAN	Panama
PRI	Puerto Rico
SLV	El Salvador
SUR	Suriname
SXM	Sint Maarten
TCA	Turks and Caicos Islands
TTO	Trinidad and Tobago
VCT	Saint Vincent and the Grenadines
VEN	Venezuela
VGB	British Virgin Islands
VIR	United States Virgin Islands

Table S3 ISO 3166-1 alpha-3 codes and countries' names for the South Asia (SA) region.

ISO3 Code	Name of Country
BGD	Bangladesh
BTN	Bhutan
IND	India
LKA	Sri Lanka
MDV	Maldives
NPL	Nepal
PAK	Pakistan

Table S4 ISO 3166-1 alpha-3 codes and countries' names for the Sub-Saharan Africa (SSA) region

ISO3 Code	Name of Country
BWA	Botswana
COM	Comoros
DJI	Djibouti
ERI	Eritrea
ETH	Ethiopia
KEN	Kenya
MDG	Madagascar
MOZ	Mozambique
MUS	Mauritius
MWI	Malawi
SOM	Somalia
SWZ	eSwatini
TZA	United Republic of Tanzania
ZAF	South Africa
ZMB	Zambia
ZWE	Zimbabwe

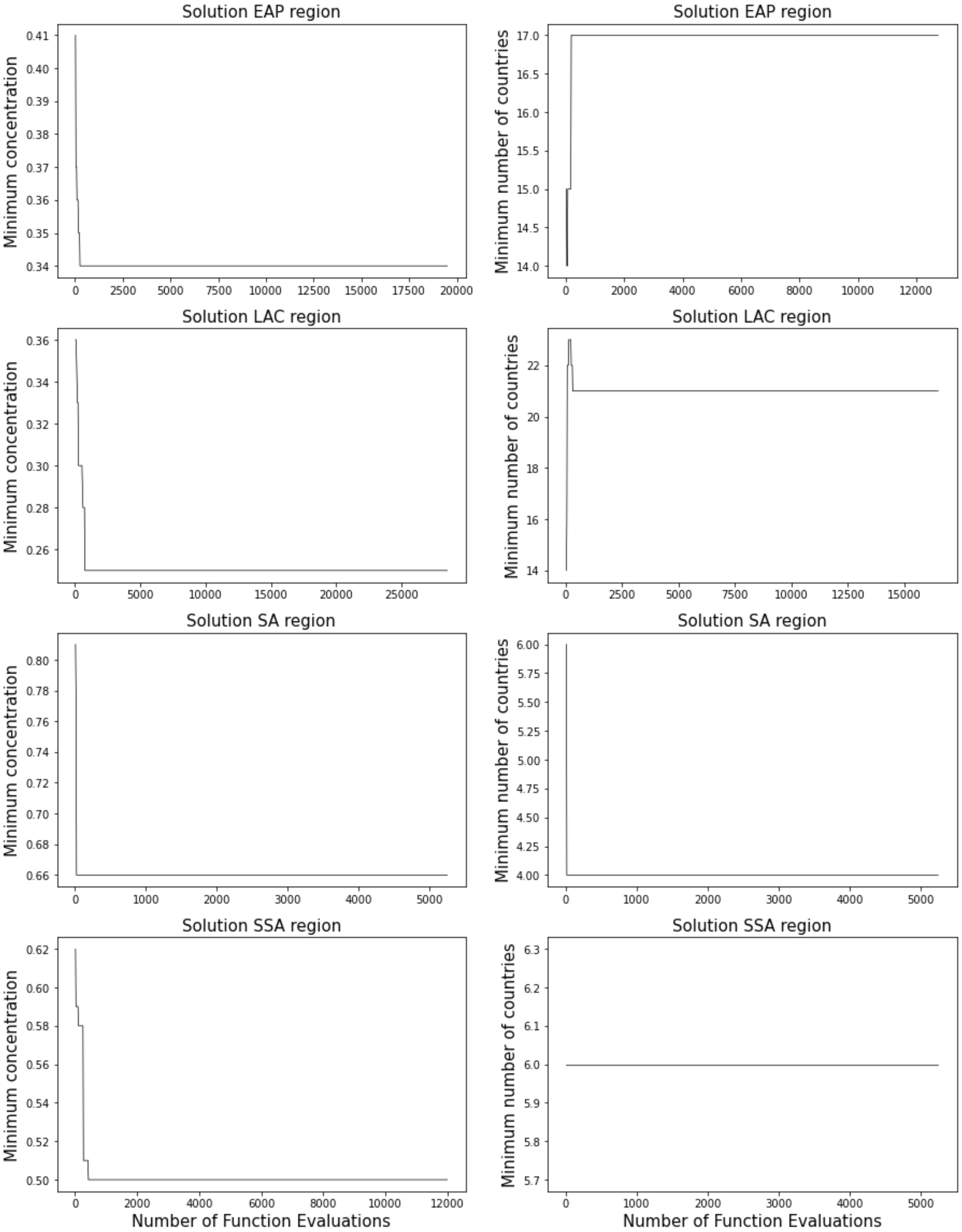

Figure S2 Convergence plots for finding the optimal regional pools in the East Asia & Pacific (EAP), Latin America & Caribbean (LAC), South Asia (SA) and Sub-Saharan Africa (SSA) regions. Left: Results from the first optimization step aimed at finding the pool with the minimum concentration. Right: Results from the second optimization step aimed at finding the smallest set of countries within the previously found pool while maintaining the minimum concentration.

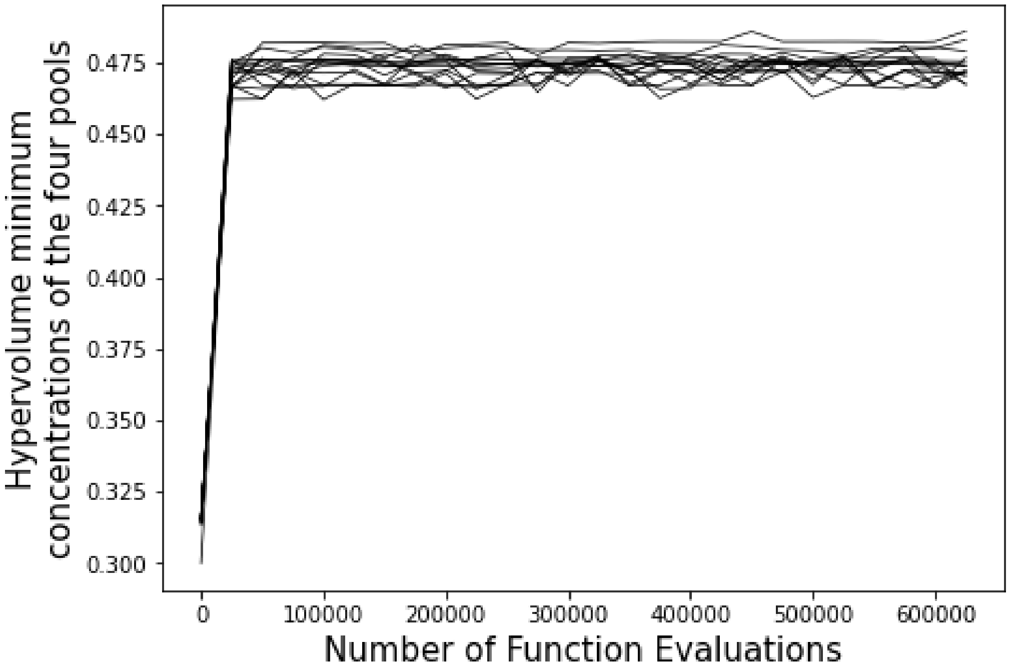

Figure S3 Convergence of the first optimization step required to find the global extensions of the regional optimal pools in the East Asia & Pacific (EAP), Latin America & Caribbean (LAC), South Asia (SA) and Sub-Saharan Africa (SSA) regions. The figure shows the hypervolume of the concentrations of the four extensions. A seed analysis is carried out and the problem is solved fifteen times.

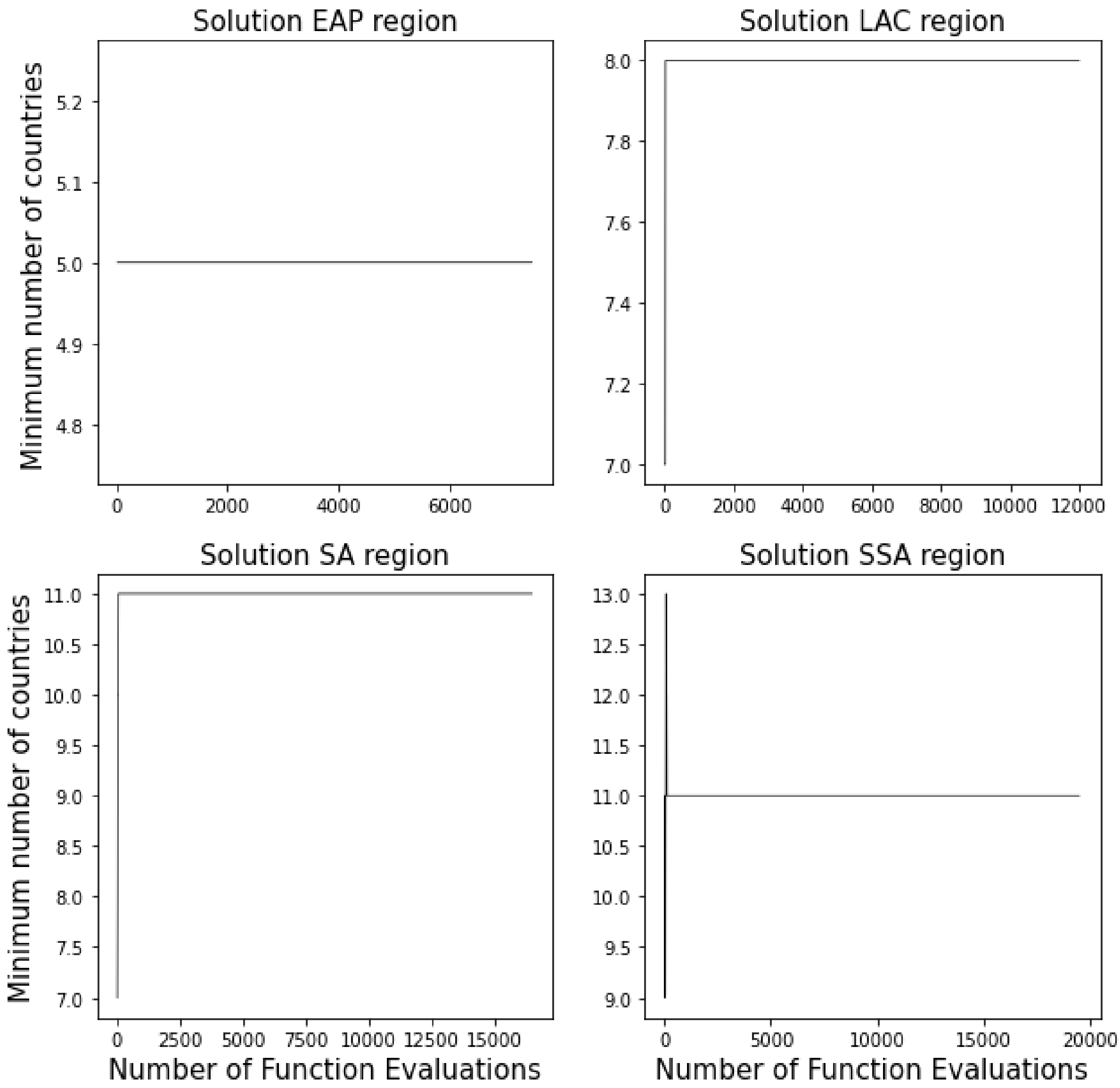

Figure S4 Convergence of the second optimization step required to find the global extensions of the regional optimal pools in the East Asia & Pacific (EAP), Latin America & Caribbean (LAC), South Asia (SA) and Sub-Saharan Africa (SSA) regions. The figure shows the smallest set of countries within the previously found extension while maintaining the minimum concentration.

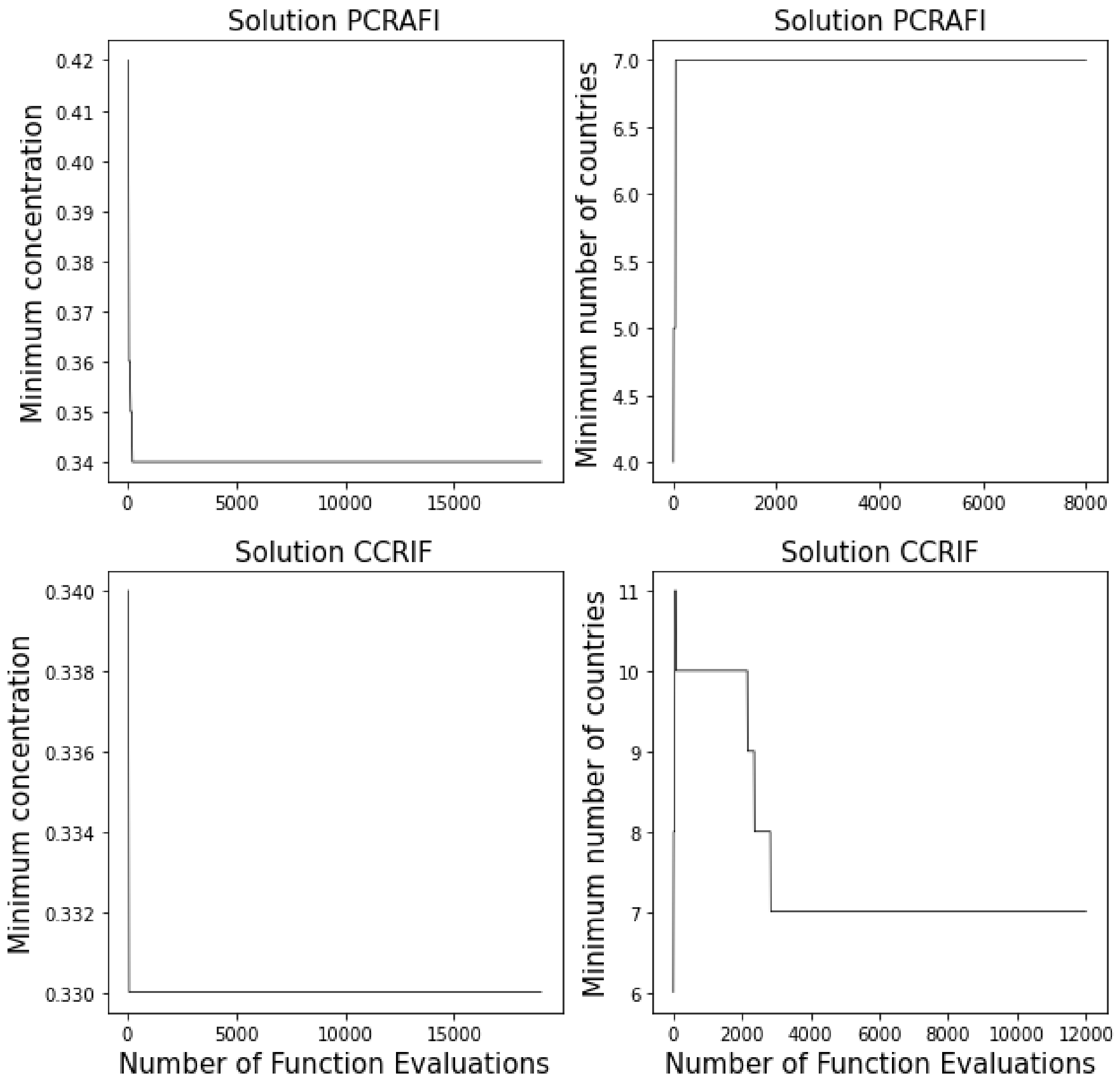

***Figure* S5** Convergence plots for finding the optimal regional extensions of PCRAFI and CCRIF. Left: Results from the first optimization step aimed at finding the extension with the minimum concentration. Right: Results from the second optimization step aimed at finding the smallest set of countries within the previously found extension while maintaining the minimum concentration.

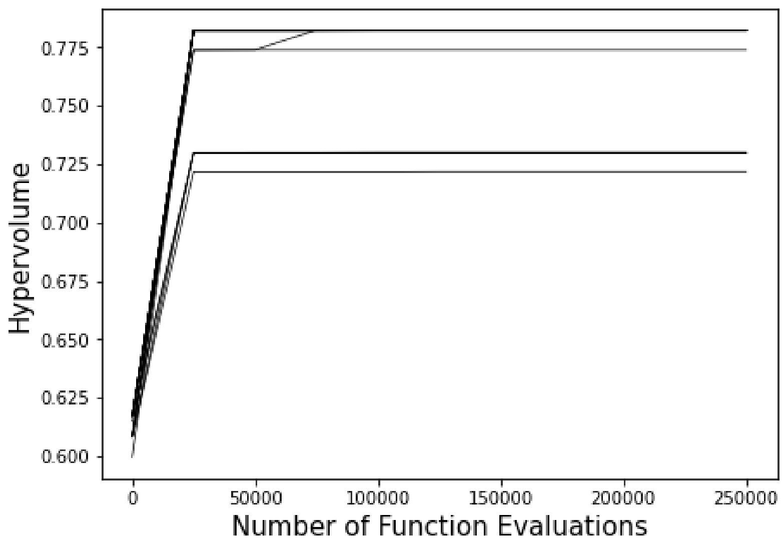

Figure S6 Convergence of the first optimization step required to find the global extensions of PCRAFI and CCRIF. The figure shows the hypervolume of the concentrations of the two extensions. A seed analysis is carried out and the problem is solved fifteen times.

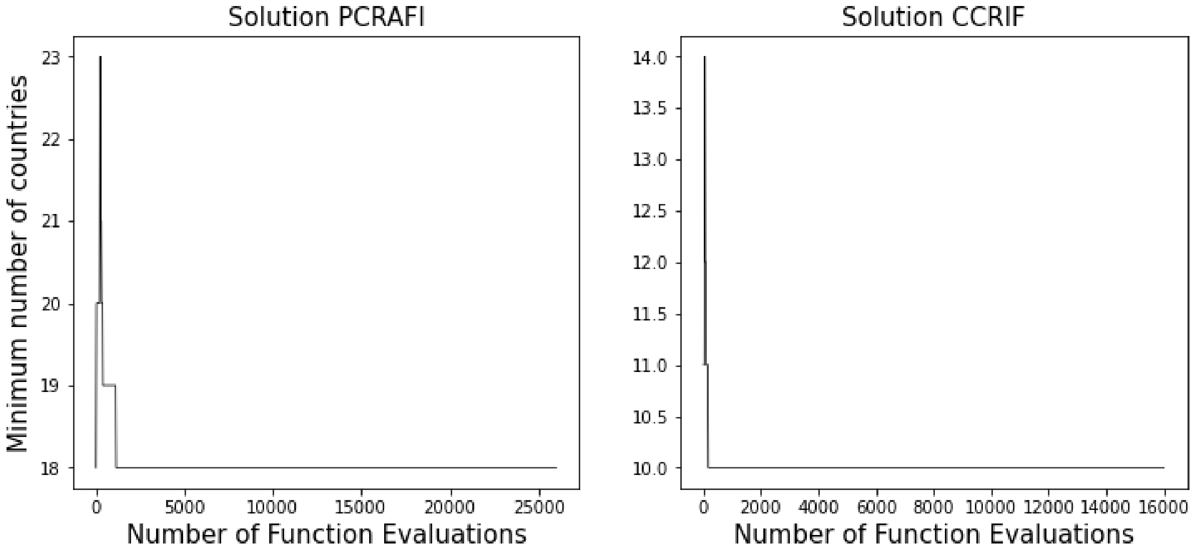

Figure S7 Convergence of the second optimization step required to find the global extensions of PCRAFI and CCRIF. The figure shows the smallest set of countries within the previously found extensions while maintaining the minimum concentration.
